# Post-thoracotomy Fibrin Glue Pleurodesis for Persisting Air Leaks: A Case Study

**DOI:** 10.7759/cureus.85280

**Published:** 2025-06-03

**Authors:** Kent W Sabatose, Chrisopher Rodriguez, C Jake Lambert Jr.

**Affiliations:** 1 Neurological Surgery, Lake Erie College of Osteopathic Medicine, Bradenton, USA; 2 Cardiothoracic Surgery, Bond Clinic, Winter Haven, USA

**Keywords:** cryoprecipitate, fibrin glue pleurodesis, persisting air leak, pleurodesis, residual pleural space, thoracotomy

## Abstract

Persistent air leaks (PALs) secondary to residual pleural spaces (RPSs) are the most common complication post-thoracotomy and positively correlate with the length of hospital stay. Among numerous treatment strategies, fibrin glue pleurodesis has the benefit of filling the RPS and can be used multiple times without anesthesia or sedation. A 71-year-old male presented with a 1 cm positron emission tomography positive nodule in the right upper lobe, who had undergone prior thoracotomy for a lung mass in 2011. Thoracotomy with wedge resection was performed with findings of diffuse lung parenchymal scarring and severe inflammatory adhesions requiring complete three-lobe decortication. Pneumothorax with a large air leak was noted postoperatively. Despite aggressive pulmonary toilet strategies, the persisting pneumothorax on postoperative day (POD) 3 required therapeutic bronchoscopy, rib resection, oversewing air leaks, topical sealant application, and three chest tube placements. Residual apical pneumothorax with PALs persisted postoperatively. Eight fibrin glue pleurodesis via a chest tube was completed over eight days, resulting in the cessation of air leak and resolution of apical pneumothorax. Fibrin glue served as a suitable pleurodesis agent in this case by filling the RPS and sealing air leaks. The body resorbs fibrin glue over time and can be performed serially to fill the RPS and avoid further surgery.

## Introduction

Persistent air leaks (PALs) are one of the most common post-thoracotomy complications, resulting in increased hospital stay and medical costs [1-3]. Residual pleural spaces (RPSs) post-thoracotomy may accentuate air leak in a positive feedback manner, making air leak resolution difficult [1]. Numerous potential treatment strategies are available to manage PALs and their issues.

These strategies can be categorized into preventative and therapeutic strategies. Intraoperatively, routine preventative strategies against PALs include pleural tenting, buttressing of staple lines to create a seal from redundant tissue from adjacent structures [2,4]. For larger defects with a greater risk of PALs and associated RPSs, intraoperative biomodification strategies can be used, such as tissue transfer flaps using the omentum, serratus anterior, latissimus dorsi, or intercostal muscle [5-7]. Perioperative discontinuation of steroid therapy and smoking cessation, although the length of abstinence required to achieve benefit is poorly defined, may also reduce the risk of PALs [8-9].

The treatment of PALs and RPSs postoperatively primarily relies on the use of chest tube(s), often placed within the apex or costodiaphragmatic recesses (pulmonary gutters). Combining chest tubes with chest drainage systems and other pulmonary toilet strategies like postural positioning, coughing, and chest percussion to drain air and blood, ventilation, and lung expansion can be seen [10]. If PALs and RPSs are unresponsive to these more conservative strategies, more invasive options may be required. Historically, sterile lucite balls (ping-pong balls) were utilized to successfully fill the RPS and reduce PALs [4]. Operative strategies for managing PAL target reduction in the thoracic cavity volume to compress the RPS. This can be accomplished through rib resection, therapeutic pneumoperitoneum, and continuous phrenic nerve block [6-7]. Pleurodesis may be utilized more commonly in smaller PAL and RPS defects and can be either chemical or mechanical pleurodesis. Mechanical pleurodesis involves surgical irritation to the pleural cavity to create scar tissue and adhesion formation, ultimately obliterating the RPS [11].

Chemical pleurodesis, utilizing sclerosing agents such as talc and bleomycin, can be effective for small-volume pneumothorax but poses a risk of chest pain, fever, acute lung injury, and empyema [11]. We created a novel pleurodesis agent, made from a mixture of thrombin and cryoprecipitate, creating a gel-like fibrin glue to fill the RPS for larger-volume pneumothorax secondary to PALs without requiring surgical intervention. Our case report serves as an example of the potential utility of serial fibrin glue pleurodesis through chest tubes for treating PALs with apical pneumothorax post-thoracotomy with wedge resection.

## Case presentation

**Figure 1:**
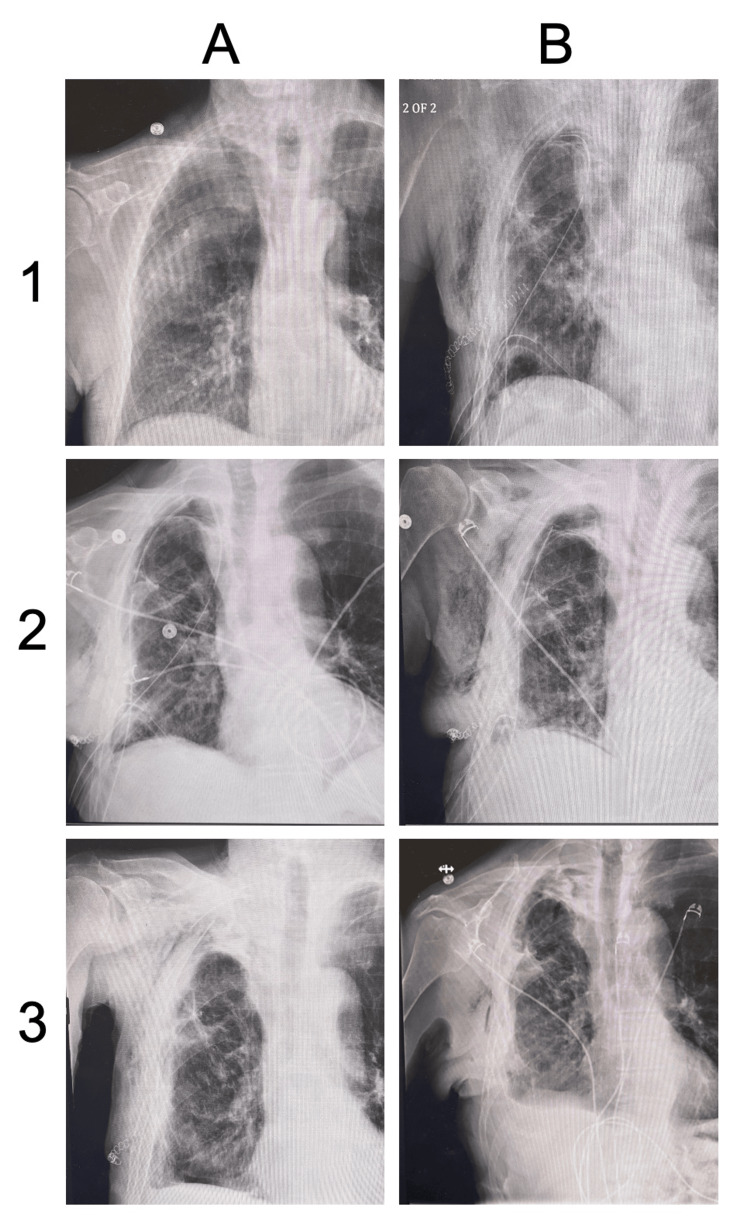
Chest radiographs of serial fibrin glue pleurodesis Plain posterior to anterior chest radiographs showing pre-surgical thoracotomy with wedge resection of the right upper lobe lung mass (1A). 1B showing postoperative changes following redo thoracotomy with a sixth rib resection and addition of three chest tubes. Serial fibrin glue pleurodesis resulting in progressive resolution of apical pneumothorax and subcutaneous emphysema (2A-3B). Note the posterior apical chest tube removal on postoperative day (POD) 6 (2B) and removal of remaining chest tubes on PODs 9 and 10 (3A and 3B, respectively). Pleurodesis material can be seen filling residual pleural space in 3B, resulting in the resolution of the air leak.

A 71-year-old man with a history of bronchiolitis obliterans organizing pneumonia (BOOP)-related right lung mass resection returned to our outpatient thoracic surgery clinic for follow-up due to a small positron emission tomography (PET)-positive right upper lobe mass (Figure 1A, Image 1A). The patient presented with adequate lung function and acceptable room air blood gases for resection and was cleared by cardiology for resection.

Medications included naproxen, aspirin 325 mg PO QD, famotidine 20 mg, tiotropium/olodaterol 2.5 mcg, acetaminophen 650 mg, folic acid 1 mg oral, methotrexate 25 mg/ml injectable, prednisone 5 mg PO QD, tizanidine 4 mg PO PRN, amlodipine 5 mg, and budesonide-formoterol. His medical history included chronic obstructive pulmonary disease (COPD), BOOP, chronic diarrhea, hyperlipidemia, rheumatoid arthritis (RA), and prostate cancer. Social history included daily ethanol use (two to four beers/day) and a 50+ pack/year smoking history.

Vitals showed a temperature of 98.5°F oral, pulse of 88 bpm, right arm blood pressure of 132/73 mmHg, weight of 66.9 kg, height of 177.8 cm, and BMI of 21.2 m/kg^2^. Lung biopsy was performed with inconclusive results, requiring mass resection.

Right thoracotomy was performed with decortication of the right upper, middle, and lower lobes due to significant adhesions and fibrosis associated with BOOP and prior lung mass resection. A right upper lobe wedge resection was performed with placement of one straight and one right-angle 36-French chest tube. Flexible fiberoptic bronchoscopy was used due to significant mucus plugging. A large bronchoscope was also required to achieve adequate mucus plug removal and lung ventilation. Complete lung expansion was achieved on POD 1.

On POD 2, a large right pneumothorax developed with collapse of the right lung despite chest tubes remaining in appropriate placement. Pulmonary toileting strategies were utilized, including right lateral recumbent with Trendelenburg positioning, with worsening right pneumothorax and a PAL noted on POD 3, leading to atrial fibrillation with rapid ventricular response of 180 bpm. The patient was stabilized with synchronous cardioversion and medically stabilized with IV diltiazem and neosynephrine prior to returning to the operating room. Resection of the right sixth rib, repeat bronchoscopy, and placement of an additional 36-French chest tube were performed to reduce the PAL and resolve the apical pneumothorax (Figure 1, Image 1B).

On POD 1 of the second procedure, the patient showed a small apical pneumothorax unchanged from the prior day, with a serosanguinous discharge and air leak noted. He was placed in right lateral decubitus posture in Trendelenburg position for two to three hours for two to three times/day. The patient continued to present with a persisting, although stable, PAL, with apical pneumothorax and subcutaneous emphysema through POD 3 despite three chest tubes in place draining approximately 350 cc of serosanguinous fluid/24 hours.

**Figure 2:**
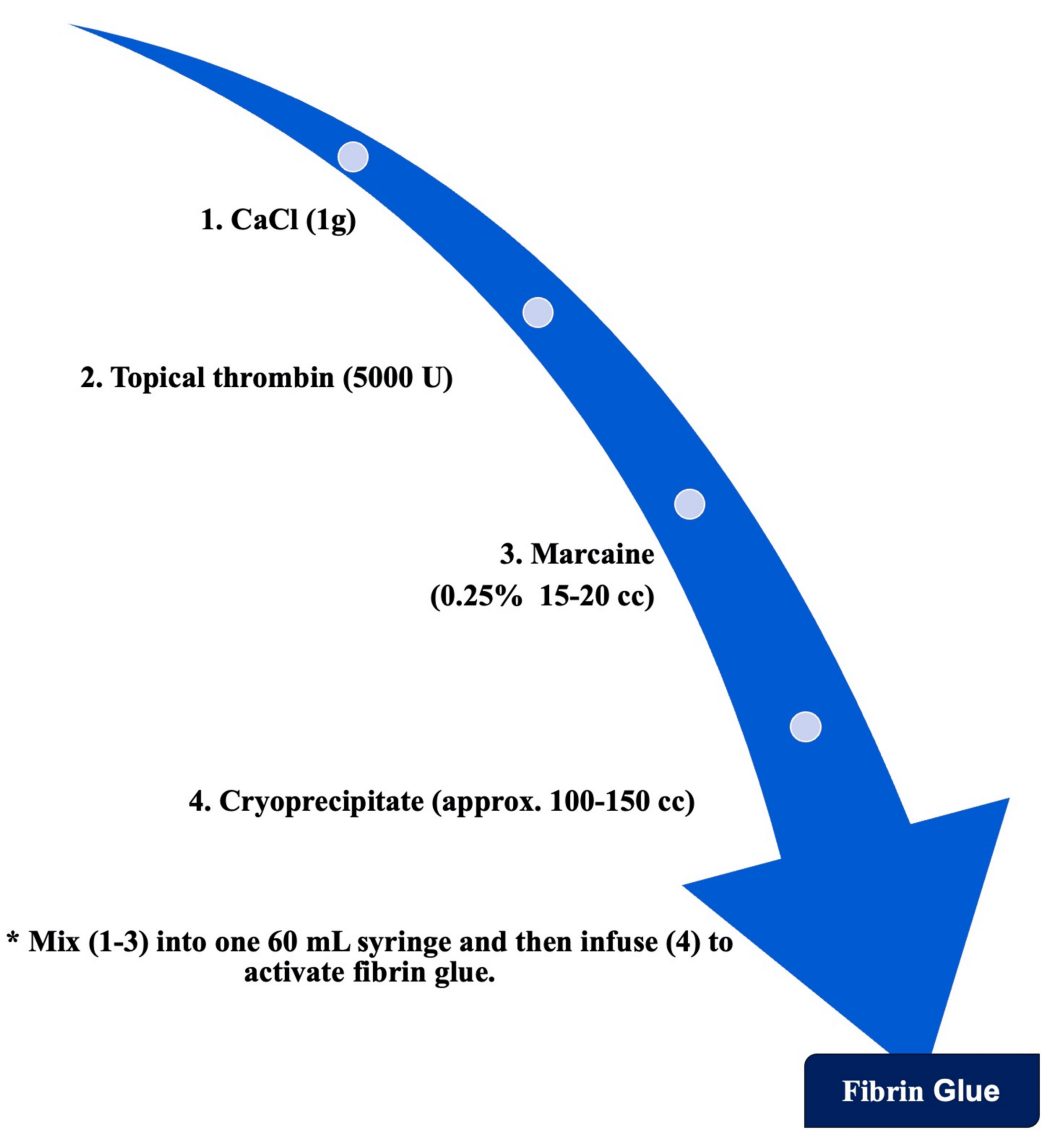
Recipe for fibrin glue

Fibrin glue pleurodesis was performed on postoperative day 3 utilizing the posterior apical chest tube for placement (Figure 1, Image 2A). The fibrin glue was made at the bedside by mixing 1 g of CaCl, 5000 U of topical thrombin, and 20 cc of 0.25% Marcaine and, lastly, combining this mixture with 150 cc of cryoprecipitate into a 60 mL syringe for injection into the desired chest tube (Figure 2). The remaining chest tubes were actively vented due to the PAL size. The posterior apical chest tube was utilized on PODs 4 and 5 for subsequent fibrin glue pleurodesis. On POD 5, the apical pneumothorax reduced in size, although persisting subcutaneous emphysema was present. The patient presented with an improved cough.

On POD 6, the posterior apical chest tube was removed, and pleurodesis was performed through the anterior-apical chest tube (Figure 1, Image 2B). Apical pneumothorax continued to reduce in size, although subcutaneous emphysema continued to increase through POD 7. Medication review discovered high-dose steroid administration. Steroid taper was immediately implemented.

Subsequent fibrin glue pleurodesis was performed on PODs 8-10 with complete resolution of apical pneumothorax and removal of the posterior-inferior and anterior-apical chest tubes on PODs 9 and 10, respectively (Figure 1, Image 3A). Evidence of an “apical cap” of pleurodesis material was noted on chest radiograph (Figure 1, Image 3B).

## Discussion

RPSs associated with PALs following thoracotomy are a common problem and pose a significant challenge in management. Risk factors associated with PALs post-thoracotomy include COPD, female sex, low forced expiratory volume in 1 second (FEV_1_), smoking history, diabetes mellitus, chronic steroid use, and marked adhesions noted intraoperatively [11-13]. Our patient presented with multiple of these risk factors, placing him at significant risk for PALs post-thoracotomy.

The American College of Chest Physicians (ACCP)'s 2001 consensus on the management of PALs recommends four days of observation of PALs for spontaneous fistula closure and surgical closure with thoracoscopy and pleurodesis for PALs lasting greater than four days. Talc or doxycycline were the ACCP's preferred pleurodesis agents in patients declining surgical intervention or in patients where surgery was contraindicated [14]. Our patient required secondary surgical biomodification through rib resection, although PALs remained unresolved. In patients with recent thoracotomy or severe illness, making surgery contraindicated, suboptimal treatment options currently exist [7,14].

Fibrin glue pleurodesis can act as a suitable pleurodesis agent in the above setting, helping to avoid further surgery. Pleurodesis with talc or bleomycin can be effective for small RPS deficits, although these agents are limited to a single treatment and require up to one month to obtain full therapeutic benefit [11]. Fibrin glue pleurodesis theoretically can be applied as many times as needed to achieve the desired residual pleural space filling.

Fibrin glue, particularly cryoprecipitate, may pose some risk for patients. Transfusion-associated circulatory overload (TACO) and transfusion-related acute lung injury (TRALI) have been documented with cryoprecipitate transfusion into the blood, with a risk estimated at one in 317,000 units of cryoprecipitate utilized [15-16]. Cryoprecipitate may also pose a risk of anaphylactic shock, pulmonary edema, intravascular hemolysis, and biliary complications, although the risk has not been established [17]. The risk profile of cryoprecipitate used for fibrin glue pleurodesis has not been investigated to our knowledge. Other pleurodesis agents, including talc and bleomycin, pose significant risks of chest pain, fever, acute lung injury, and empyema, which can limit the utilization of these procedures for unstable patients [11]. In our experience, fibrin glue pleurodesis caused minimal to no pain for our patient and can be performed without sedation in the ICU. We anticipate the fibrin glue will resorb into the body over time without producing scar formation.

Other newer strategies for addressing PALs include continuous phrenic nerve block and therapeutic pneumoperitoneum [18-20]. These techniques address PALs indirectly by compressing the thoracic cavity to reduce the RPS deficit. Blood patch techniques have also been utilized to seal off areas of PALs, although this strategy has no space-filling property [21]. Fibrin glue pleurodesis can achieve both RPS filling and sealing of PALs.

## Conclusions

To the best of our knowledge, this case study is the first on the use of fibrin glue as a pleurodesis agent to fill RPSs and stop PALs. In our experience, fibrin glue pleurodesis can be a suitable strategy for PALs post-thoracotomy and is well tolerated by the patient. The benefits of fibrin glue as a pleurodesis agent include its ability to be absorbed by the body and potential for multiple applications for additive effect without the need for anesthesia or sedation. The ACCP's guidelines reserve talc or doxycycline pleurodesis for patients unable to undergo surgical intervention or who refuse surgery, although fibrin glue pleurodesis may serve as an additional strategy for PALs greater than four days for this patient population. Fibrin glue pleurodesis, due to its ability to treat larger volume pleural space deficits compared to other pleurodesis agents, might mitigate the need for additional surgery to correct PALs. Despite the above benefits of fibrin glue pleurodesis, limited to no literature exists on the response of the body to fibrin glue within the pleural space. Prospective studies with larger sample sizes are needed to investigate the efficacy of fibrin glue pleurodesis and the potential adverse effects of this novel pleurodesis agent.
